# Elevated acute-phase plasma levels of S100A12 [EN-RAGE] are associated with vascular recurrence after ischemic stroke

**DOI:** 10.1093/esj/23969873251384439

**Published:** 2026-01-01

**Authors:** Björn Granelli, Annelie Angerfors, Sofia Furutjäll, Hanh Nguyen Larsson, Cecilia Brännmark, Björn Andersson, Tara M Stanne, Christina Jern

**Affiliations:** Institute of Biomedicine, Department of Laboratory Medicine, Sahlgrenska Academy, University of Gothenburg, Gothenburg, Sweden; Institute of Biomedicine, Department of Laboratory Medicine, Sahlgrenska Academy, University of Gothenburg, Gothenburg, Sweden; Institute of Biomedicine, Department of Laboratory Medicine, Sahlgrenska Academy, University of Gothenburg, Gothenburg, Sweden; Institute of Biomedicine, Department of Laboratory Medicine, Sahlgrenska Academy, University of Gothenburg, Gothenburg, Sweden; Department of Medicine, Geriatrics and Emergency Medicine, Sahlgrenska University Hospital/Östra Sjukhuset, Gothenburg, Sweden; Bioinformatics and Data Center, Core Facilities, Sahlgrenska Academy, University of Gothenburg, Gothenburg, Sweden; Institute of Biomedicine, Department of Laboratory Medicine, Sahlgrenska Academy, University of Gothenburg, Gothenburg, Sweden; Region Västra Götaland, Department of Clinical Genetics and Genomics, Sahlgrenska University Hospital, Gothenburg, Sweden; Institute of Biomedicine, Department of Laboratory Medicine, Sahlgrenska Academy, University of Gothenburg, Gothenburg, Sweden; Region Västra Götaland, Department of Clinical Genetics and Genomics, Sahlgrenska University Hospital, Gothenburg, Sweden

**Keywords:** Ischemic stroke, proteomics, S100A12 [EN-RAGE] protein, brain ischemia, stroke

## Abstract

**Introduction:**

Despite modern secondary prevention the risk of recurrent vascular events in ischemic stroke remains substantial, and high-sensitivity C-reactive protein (hsCRP) and interleukin-6 (IL-6) are associated with vascular recurrence. This study aims to investigate whether other proteins in the inflammatory cascade could serve as better predictive biomarkers.

**Patients and methods:**

The discovery cohort comprised 559 ischemic stroke cases from *SAHLSIS* (age 18–69, median 58 years) with a median follow-up of 14.7 years. Acute-phase plasma levels of 65 inflammation-related proteins were assessed using the Olink Inflammation panel. Replication was sought using 502 cases from *SAHLSIS2* (age 18–98, median 68 years) with a median follow-up of 3.6 years. Associations between proteins and recurrent major adverse cardiovascular events (MACE) and recurrent stroke were explored with Cox regression. For MACE in *SAHLSIS*, exploratory analyses stratified by etiologic subtype were performed. Analyses were adjusted for vascular risk factors and statin status.

**Results:**

In *SAHLSIS*, S100A12 was independently associated with recurrent MACE (adjusted hazard ratio (HR), 1.27 [95% confidence interval 1.10–1.45] per doubling of protein level) and stroke (adjusted HR 1.21 [1.01–1.45]). In *SAHLSIS2*, the associations for S100A12 replicated (adjusted HR, recurrent MACE 1.25 [1.06–1.48] and stroke 1.35 [1.10–1.66]). Results from the exploratory analyses identified several proteins displaying subtype-specific associations.

**Discussion:**

We identified S100A12 as a potential novel blood biomarker of vascular recurrence after ischemic stroke, and the results indicate that there are subtype-specific protein associations to recurrent MACE warranting further investigation.

## Graphical Abstract

**Graphical Abstract:**
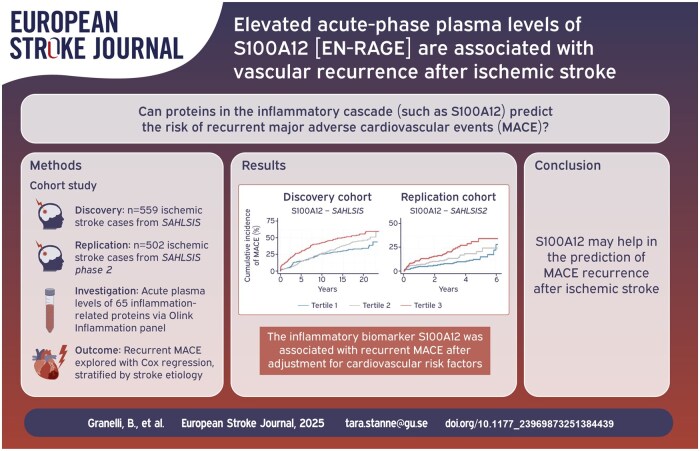

## Introduction

Ischemic stroke survivors face a high risk of recurrent major adverse cardiovascular events (MACE) such as recurrent stroke and myocardial infarction, and about one in four stroke patients suffer from a recurrent vascular event.^1,2^ Results from both the *Sahlgrenska Academy Study on Ischemic Stroke* (*SAHLSIS*) and the *Follow-Up of Transient ischemic attack and stroke patients and Unelucidated Risk factor Evaluation* (*FUTURE*) study, which included adult patients with ischemic stroke under the age of 70 and 50 years, respectively, show that recurrent MACE are common also among younger ischemic stroke survivors and that the risk remains elevated for many years.^3,4^ As the stroke fatality rate has declined^5^ and the incidence of ischemic stroke is increasing in the young,^6^ there will in the future be a larger number of stroke survivors facing the risk of vascular recurrence, which demonstrates the urgent need to develop new secondary prevention strategies for stroke.

Inflammation is known to play a role in the pathophysiology of ischemic stroke^7^ and is a well-established contributor to the pathophysiology of atherosclerosis.^8,9^ Emerging evidence suggests that inflammation may play a pathophysiological role in stroke caused by cardioembolism and small vessel disease,^10^ emphasizing the role of inflammation across various ischemic stroke subtypes. Several studies also suggest that a pro-inflammatory state increases the risk of recurrent MACE and stroke following ischemic stroke. The most studied circulating protein biomarkers in this regard are high-sensitivity CRP (hsCRP) and interleukin 6 (IL-6).^10–12^ We hypothesize that other inflammation-related protein biomarkers, especially proteins more upstream in the inflammation cascade, could serve as better predictors of vascular recurrence after ischemic stroke and/or as more precise therapeutic targets for secondary prevention. Considering this, we here aimed to broadly profile inflammation-related proteins and investigate their associations to long-term vascular recurrence in a prospective cohort of adult patients with acute ischemic stroke before 70 years of age. We then sought replication in a cohort of ischemic stroke adults of all ages.

## Methods

### Standard protocol approvals

This study was conducted in adherence to the guidelines for Strengthening the Reporting of Observational Studies in Epidemiology, STROBE (see Supplemental Material). Written informed consent or consultant consent from next-of-kin were obtained for all participants prior to enrollment. This study was approved by the Regional Ethics Review Board in Gothenburg, Sweden (registration numbers: 469-99, T553-03, 413-04, T665-07, T586-13, 823-13, T1110-16).

### Study population and baseline characteristics

Study participants for the discovery cohort were from the hospital-based observational cohort study the *Sahlgrenska Academy Study on Ischemic Stroke* (*SAHLSIS*), and replication was sought from *SAHLSIS phase 2* (*SAHLSIS2*). Both studies have been described in detail.^13,14^ In brief, adult ischemic stroke patients with first-ever or recurrent acute ischemic stroke were recruited at stroke units in Western Sweden. In *SAHLSIS*, patients with ischemic stroke before 70 years of age were included 1998–2003, and in *SAHLSIS2*, ischemic stroke patients of all ages were included 2015–2020. Acute ischemic stroke was defined as an episode of focal brain dysfunction with acute onset, lasting >24 h, and of presumed vascular cause with no signs of hemorrhage or another cause on neuroimaging. All study participants underwent imaging of the brain (computed tomography and/or magnetic resonance imaging) as part of the clinical routine investigation. If clinically indicated, patients were also evaluated by angiography and perfusion measures. Participants underwent additional work-up according to national guidelines and were excluded if further evaluation showed another etiology than ischemic stroke. Etiologic subtypes of ischemic stroke were classified according to the Trial of Org 10172 in Acute Stroke Treatment (TOAST) criteria with minor modifications as described^13,15^ into large artery atherosclerosis (LAA), small artery occlusion (SAO), cardioembolic (CE) stroke, cryptogenic stroke (defined as no cause identified cause despite a complete evaluation), other determined causes, and undetermined stroke (defined either as incomplete evaluation or two or more causes identified).

In *SAHLSIS*, complete information on vascular risk factors was registered at 3-month follow-up by examinations and in structured questionnaires as described.^13^ In brief, hypertension was defined as either systolic blood pressure ⩾ 160 mmHg, diastolic blood pressure ⩾ 90 mmHg or pharmacological treatment for hypertension. Diabetes was defined either from blood samples as fasting plasma glucose ⩾ 7.0 mmol/l, fasting blood glucose ⩾ 6.1 mmol/l or as either dietary or pharmacological treatment for diabetes. Hyperlipidemia was defined as either total fasting serum cholesterol ⩾ 5.0 mmol/l, low density lipoprotein ⩾ 3 mmol/l or pharmacological treatment for hyperlipidemia. Smoking was defined as current smoking or cessation of smoking within the last year prior to inclusion. Prescribed drugs, including statin treatment, at discharge were also registered. In *SAHLSIS2*, data on diabetes mellitus, hypertension, statin treatment at discharge, atrial fibrillation, and smoking were retrieved from the national quality register for stroke, Riksstroke.^14,16^ In *SAHLSIS*, acute stroke severity was defined according to the Scandinavian Stroke Scale (SSS). The score reflecting the highest neurological deficit within 7 days of hospital admission was used and converted to the international and more commonly used NIH Stroke Scale (NIHSS) score using an established algorithm.^17^ Of note, during the inclusion period of *SAHLSIS*, acute revascularization therapy was not yet part of routine clinical practice. In *SAHLSIS2* stroke severity was defined using the NIHSS score either at admission for patients who did not undergo revascularization therapy or 24 h after revascularization therapy.

### Blood sampling

Venous blood samples were drawn in the acute phase (median [interquartile range, IQR] 4 [3-6] and 2 [2-4] days after index stroke in *SAHLSIS* and *SAHLSIS2*, respectively). All samples were collected between 8:30 a.m. and 10:30 a.m. after overnight fast. Blood was collected in tubes containing 10% by volume ethylenediaminetetraacetic acid (EDTA). Plasma was isolated within 2 h by centrifugation at 2000×*g* at 4°C for 20 min, aliquoted, and stored at −80°C pending analysis.

### Measurement of proteins levels

In *SAHLSIS*, plasma levels of inflammatory-related proteins were measured using the highly sensitive and specific Proximity Extension Assay (PEA) using the Inflammation I panel (Olink Proteomics, Uppsala, Sweden) as described,^18^ which includes IL-6. Analyses were performed according to the manufacturer’s protocol by board-certified laboratory technicians blinded to clinical information. As described, a total of 65 proteins showed the prespecified call rate > 80%.^18^ Plasma protein levels were measured as Normalized Protein eXpression (NPX) values, which is a relative quantification given on a log2 scale. Plasma samples in the acute phase were missing for 39 cases and information from national registers were missing for 2 patients, giving us a total of 559 patients.^18^ We previously measured serum levels of hsCRP in *SAHLSIS* using an immunoassay technique as described,^19^ and we therefore present these in the current study for comparison also on a log2 scale.

*SAHLSIS2* was used for replication of findings for proteins that were independently associated with recurrent MACE and/or stroke. Data on plasma levels of these proteins were retrieved from measurements using the PEA Olink Explore (*n* = 200) or Olink Explore HT (*n* = 302). All analyses were performed by board-certified laboratory technicians blinded to the clinical information at either Olink Proteomics AB (Olink Explore) or at the Affinity Proteomics unit at the National Genomic Infrastructure Uppsala (NGI), Science for Life Laboratory (SciLifeLab; Olink Explore HT). As in *SAHLSIS*, plasma protein levels were measured as NPX values.

### Outcome assessment

We assessed all participants from both *SAHLSIS* and *SAHLSIS2* regarding recurrent MACE until December 31, 2021 using national registers as described in detail elsewhere.^3^ MACE was pre-defined as any recurrent stroke, major coronary event or death due to a vascular cause, whatever happened first. Patients were censored at time of death if the cause of death was not one of the predefined endpoints, and a small number of patients (*n* = 5) were also censored when moving abroad as described.^4^ Data regarding non-fatal vascular events was gathered from the National Hospital Discharge Registry and data regarding deaths from the Swedish National Cause of Death Registry. The following ICD codes were used to screen for fatal or nonfatal recurrent stroke, I60.0-I68.8; fatal or nonfatal coronary events, I21.0-I22.9; and other vascular deaths, I10-I28, I42-I50, I69-I89, and F01.^3^ For coronary events recorded until the end of 2012, we also screened codes for percutaneous interventions or coronary bypass grafting, that is the codes FNG00-FNG96, FNA00-FNF96, and FNH00-FNW98.^3^ When possible, all recorded diagnoses were verified by review of the patient’s medical records. In cases where the medical record corresponding to the recorded diagnosis could not be found, the diagnosis was included in the study if it was listed as the primary diagnosis or primary procedure for the relevant hospital admission. Classification of outcomes was performed blind to biomarker results.

### Statistical analyses

Baseline characteristics were compared using Mann-Whitney for continuous variables, and by Chi-square tests for categorical variables. Our primary endpoint was recurrent MACE, and our secondary endpoint was recurrent stroke. Univariable and multivariable Cox proportional hazards regression models were used to estimate hazard ratios and 95% confidence intervals ([HRs and 95% CI]) for each biomarker for both endpoints. As the NPX value is given on a log2 scale, the yielded HR corresponds to the predicted increased risk of suffering an event per each doubling of the protein level. The proportional hazards assumption was analyzed by Schoenfeld’s tests. For *SAHLSIS*, analyses were adjusted for clinical factors found to be significantly different (*p* < 0.05) between the MACE and non-MACE groups based on chi-square or Mann-Whitney. These factors included age, diabetes mellitus, hypertension, and previous coronary artery disease (CAD). Additional adjustments were made for sex and the day of blood draw due to their plausible influence on protein levels and statin treatment as it has been linked to inflammatory protein levels and vascular outcomes. To correct for multiple testing, false discovery rate (FDR) was applied. It is, however, of note that many of these proteins are highly correlated,^18^ making an adjustment for 66 independent comparisons arguably too stringent. Therefore, we also report nominal associations (i.e. *p-value* < 0.05 and a FDR adjusted *p*-value > 0.05).

For proteins nominally associated with MACE in univariable and/or multivariable analyses in *SAHLSIS*, we performed sensitivity analyses including: (1) only cases who experienced a MACE before the median time to event, that is 6 years of follow-up, as most previous studies have shorter follow-up times and thus a lower percentage of MACE compared to *SAHLSIS*; and (2) only first-ever strokes, as previous strokes have been associated with changes in inflammation protein concentrations in the long-term after stroke.^18^

Replication of proteins associated with recurrent MACE and/or stroke at the nominal or significant threshold in multivariable analyses in *SAHLSIS* (i.e. CD-6 and S100A12 [EN-RAGE]) were sought in *SAHLSIS2* using univariable and multivariable Cox regressions. The multivariable model was adjusted for age, sex, day of blood draw, diabetes mellitus, hypertension, atrial fibrillation, and statin treatment. Data on previous CAD was unavailable for this cohort.

For the top proteins, participants were stratified into tertiles based on protein levels and Kaplan-Meier curves were used to visualize plasma protein associations with cumulative recurrent MACE or stroke (unadjusted associations). Standard log-rank tests were used to assess differences in cumulative incidence of recurrent MACE or stroke between the survival curves for each tertile, with *p*-values < 0.05 considered significant. Associations with recurrent vascular events were also analyzed per protein tertile in uni- and multivariable Cox regression analyses to assess dose-response relationships, with the bottom tertile 1 (T1) as reference.

Finally, in *SAHLSIS*, we also performed exploratory analyses for MACE stratified by the four main TOAST subtypes LAA, CE, SAO, and cryptogenic stroke.

Data were analyzed using R Version 4.4.0 (R Foundation for Statistical Computing, Vienna, Austria), survival package (version 3.7.0) for survival analyses and Cox-regressions. For visualization the following packages were used ggplot2 (version 3.5.1), survminer (version 0.4.9), ggfortify version (0.4.17), gridExtra (version 2.3), gtsummary (version 2.0.3) and gt (version 0.11.1).

### Data availability

Anonymized data will be shared upon reasonable request, provided data transfer agrees with EU legislation on the general data protection regulation and with decisions by the Ethical Review Board of Sweden and the University of Gothenburg, the latter which should be regulated in a data transfer agreement.

## Results

Among the 559 study participants from *SAHLSIS*, 248 (44%) experienced a recurrent MACE during the median follow-up of 14.7 years (7760 patient-years). Of these, the first event was a fatal/nonfatal recurrent stroke in 129, a fatal/nonfatal major coronary event in 77, or other cause of vascular death in 42. Among the 502 cases in *SAHLSIS2*, 78 (16%) experienced a recurrent MACE during the median follow-up of 3.6 years (1790 patient-years), of which 53 were a recurrent stroke, 10 were a major coronary event, and 15 were vascular death of other cause. Baseline characteristics for these groups are summarized in Table 1. Participants that experienced a recurrent MACE were generally older, with a higher percentage of diabetes mellitus, hypertension, previous CAD compared to participants with no events during follow-up (Table 1).

**Table 1.** Baseline characteristics for *SAHLSIS* and *SAHLSIS2*.

Variable	*SAHLSIS*					*SAHLSIS2*				
	All	MACE	Recurrent stroke	No event	*p*-Value*	All	MACE	Recurrent stroke	No event	*p*-Value*
	*n* = 559	*n* = 248	*n* = 149	*n* = 311		*n* = 502	*n* = 78	*n* = 53	*n* = 424	
Age, years, median [IQR]	58 [52–64]	61 [55–65]	61 [55–65]	56 [49–61]	<0.001	68 [56–77]	72 [64–80]	70 [60–78]	68 [55–76]	0.002
Male sex, *n* (%)	354 (63)	166 (67)	95 (64)	188 (61)	n.s.	305 (61)	48 (62)	33 (62)	257 (61)	n.s.
Day of blood draw, median [IQR]	4 [3–6]	4 [3–6]	4 [3–6]	5 [3–7]	n.s.	4 [2–6]	3 [2–6]	3 [2–5]	4 [2–6]	n.s.
Diabetes mellitus, *n* (%)	104 (19)	65 (26)	32 (22)	39 (13)	<0.001	65 (13)	21 (27)	13 (25)	44 (10)	<0.001
Hypertension, *n* (%)	334 (60)	167 (67)	101 (68)	167 (54)	<0.001	215 (43)	54 (69)	37 (70)	161 (38)	<0.001
Current smoker, *n* (%)	217 (39)	104 (42)	63 (42)	113 (36)	n.s.	55 (12)	10 (14)	8 (15)	45 (11)	n.s.
Statin treatment^†^, *n* (%)	186 (33)	87 (36)	47 (32)	99 (32)	n.s.	405 (81)	59 (78)	43 (84)	346 (82)	n.s.
Atrial fibrillation, *n* (%)	62 (11)	31 (12)	19 (13)	31 (10)	n.s.	111 (22)	18 (23)	7 (13)	93 (22)	n.s.
Previous CAD, *n* (%)	73 (13)	49 (20)	22 (15)	24 (8)	<0.001	-	-	-	-	-
NIHSS score, median [IQR]	3 [2–7]	3 [2–8]	4 [2–8]	2 [1–6]	0.043	2 [1–6]	4 [1–7]	3 [0–6]	2 [1–6]	n.s.
MACE before 2184 days, *n*	559	132	90	427	n.a.	-	-	-	-	-
First ever stroke, *n*	484	200	121	284	n.a.	475	71	50	389	n.a.

Data are shown as median and interquartile range (IQR), mean and standard deviation (SD), or number (*n*) and percentage.

^*^Mann-Whitney or Chi-square test comparing MACE and no-event.

^†^Statin treatment at hospital discharge.

### Primary analysis: Associations between plasma protein levels and recurrent MACE

**Figure 1.**
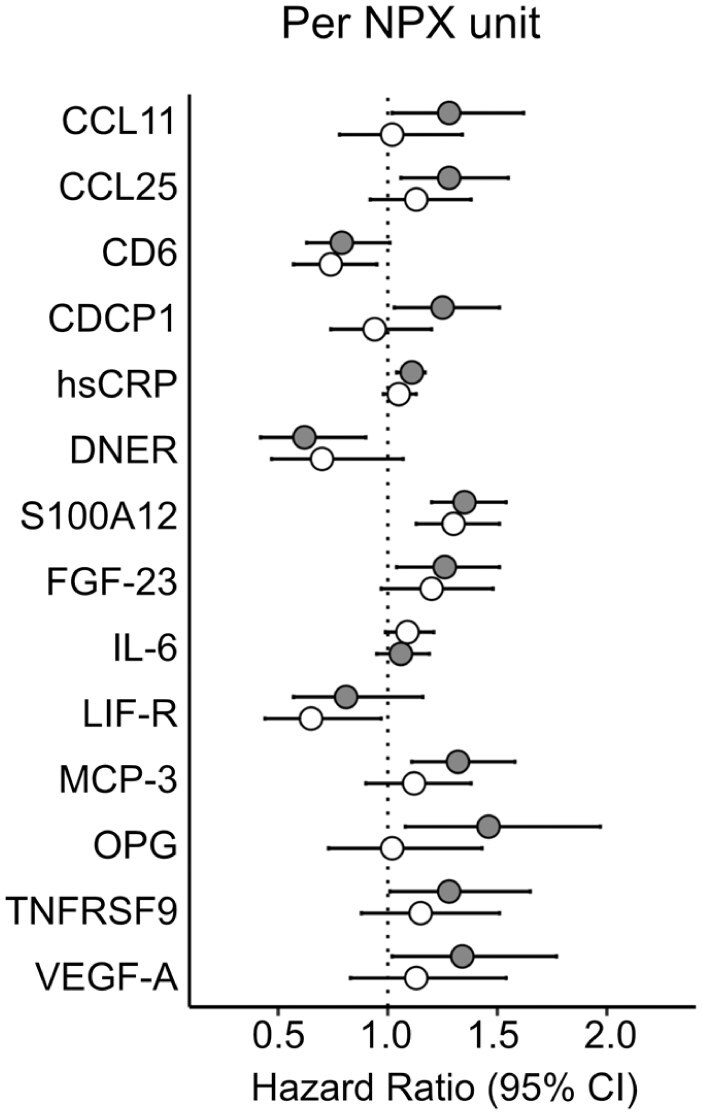
Forest plots of associations between plasma proteins and recurrent MACE in univariable and multivariable Cox regression models. Hazard Ratios per doubling of protein level with 95% confidence intervals. Gray, unadjusted. White, adjusted for age, sex, day of blood draw, hypertension, diabetes mellitus, previous coronary artery disease, and statin treatment.

Results for proteins that showed significant or nominal associations to MACE in univariable and/or multivariable Cox regression analyses in *SAHLSIS* are shown in Figure 1. For comparison with previous studies, results on IL-6 are also displayed although it was not significantly associated with MACE. Results for all 66 proteins are available in the Online Supplement (Supplemental Table S1). In the univariable analyses, statistically significant associations were observed for S100A12 (hazard ratio (HR), 95% confidence interval [95% CI] per doubling of protein level (i.e. NPX unit): 1.36 [1.20–1.53], FDR = 6 × 10^−5^), hsCRP (HR: 1.10 [1.04–1.18], FDR = 0.04), and MCP-3 (HR: 1.32 [1.11–1.58], FDR = 0.04) and nominal associations for a further 8 proteins (CCL11, CCL25, CDCP1, DNER, FGF-23, OPG [alias TNFRSF11B], TNFRSF9, and VEGF-A). In the multivariable analyses, elevated S100A12 remained associated with an increased risk of MACE (adjusted HR [95% CI]: 1.26 [1.10–1.45], *p* = 0.0008 per NPX unit), though this was just below FDR-corrected significance (FDR = 0.052). Elevated CD6 was nominally associated with decreased risk of MACE (adjusted HR: 0.76 [0.59–0.97], *p* = 0.031).

**Figure 2.**
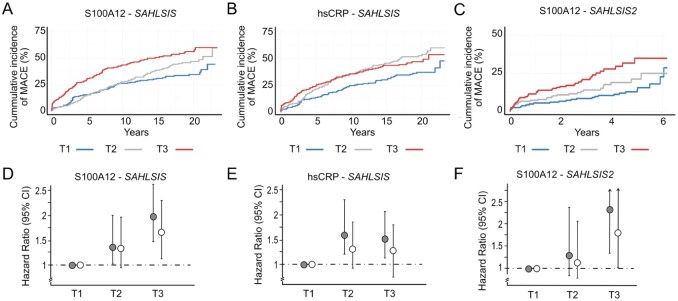
Per tertile analyses for recurrent MACE in the discovery cohort (*SAHLSIS*) and the replication cohort (*SAHLSIS2*). (a–c) Kaplan-Meier curves showing cumulative incidence of MACE. Participants were grouped into tertiles of each protein. Blue, tertile 1 (T1, lowest protein levels); gray, tertile 2 (T2); red, tertile 3 (T3, highest protein levels). (d–f) Forest plots of per tertile analyses, with the lowest tertile (T1) as reference. Gray, unadjusted. White, adjusted for vascular risk factors and statin treatment.

We next assessed differences in the cumulative incidence of recurrent MACE per protein tertile (Figure 2). Eight proteins demonstrated a difference in survival curves between protein tertiles with highest cumulative incidence of MACE in the highest tertile (standard log-rank *p* < 0.05: S100A12, CCL11, CCL25, CDCP1, hsCRP, MCP-3, OPG, and VEGF-A). Kaplan-Meier plots for S100A12 tertiles are displayed in Figure 2(a), and for comparison the corresponding plots for hsCRP tertiles are displayed in Figure 2(b). The plots for the remaining six proteins are available in the Online Supplement (Supplemental Figure S1). On per tertile Cox regression analysis, S100A12 showed a dose-response association in both univariable and multivariable analysis with the highest risk for recurrent MACE in highest protein tertile (adjusted HR 1.65 [1.18–2.30], T3 vs T1; Figure 2(d)). No dose-response was observed for hsCRP (Figure 2(e)).

In our older-aged replication cohort with shorter follow-up, *SAHLSIS2*, S100A12 was associated with MACE in both univariable (HR [95% CI]: 1.29 [1.11–1.51], *p* = 0.001 per NPX unit) and multivariable analyses (adjusted HR, 1.25 [1.06–1.48], *p* = 0.008). As depicted in Figure 2(c), we also replicated a difference in survival curves between protein tertiles of S100A12, with highest cumulative incidence of MACE in the highest tertile (standard log-rank *p* = 0.0013). Compared with patients in the bottom tertile (T1) of S100A12, patients in the top tertile (T3) had a 77% increased risk of recurrent MACE after adjustment for vascular risk factors and statin treatment (adjusted HR 1.77 [1.00–3.14], T3 vs T1; Figure 2(f)). The association for CD-6 did not replicate in *SAHLSIS2* (adjusted HR: 1.07 [0.68–1.70].

### Sensitivity analyses for MACE

To enable comparisons with other studies with shorter follow-up times, sensitivity analyses were performed in *SAHLSIS* for proteins showing significant or nominal associations to MACE restricting the analysis to 6-year follow-up (i.e. before median). In univariable analyses, 7 proteins were associated with MACE (HR [95% CI] CCL11, 1.38 [1.0–1.9]; hsCRP, 1.16 [1.06–1.26]; FGF-23, 1.41 [1.12–1.77]; IL-6, 1.16 [1.02–1.32]; MCP-3, 1.36 [1.07–1.72]), S100A12, 1.45 [1.23–1.70]; and TNFRSF9, 1.49 [1.06–2.09]). Only S100A12 remained associated in multivariable analyses (adjusted HR [95% CI]: 1.34 [1.11–1.61], *p* = 0.003 per NPX unit). For details, see the Online Supplemental Table S2.

As ischemic stroke has been associated with longitudinal changes in inflammation protein concentrations, we also performed sensitivity analyses including only cases who had a first-ever stroke as their index event. S100A12 was associated with MACE in both uni- and multivariable analyses in first-ever strokes (adjusted HR [95% CI]: 1.29 [1.11–1.51], *p* = 0.001 per NPX unit). Details for the remaining proteins can be found in the Online Supplemental Table S3.

### Secondary analysis: Associations between plasma protein levels and recurrent stroke

**Figure 3.**
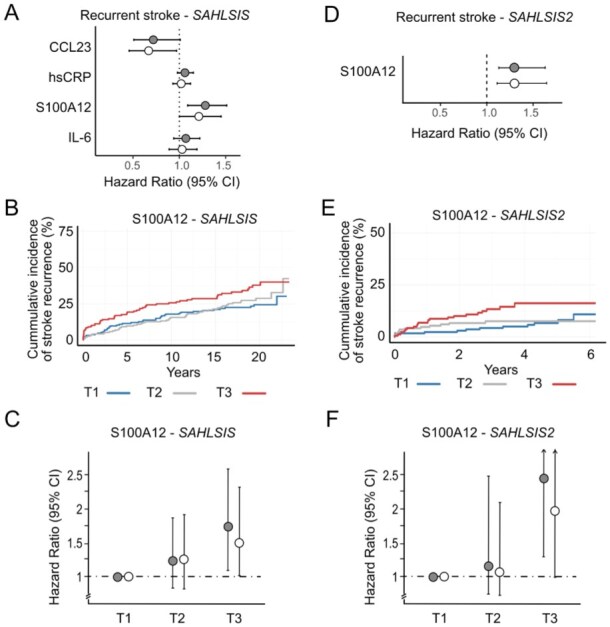
Elevated S100A12 is associated with increased risk of recurrent stroke. (a and d) Forest plots of associations between plasma proteins and recurrent stroke in univariable and multivariable Cox regression models in *SAHLSIS* and *SAHLSIS2*, respectively. Plots shows the HRs with 95% confidence intervals per doubling of protein level. Gray, unadjusted. White, adjusted for vascular risk factors and statin treatment. (b and e) Kaplan-Meier curves showing cumulative incidence of stroke recurrence for protein tertiles of S100A12 in *SAHLSIS* and *SAHLSIS2*, respectively. Participants were grouped into tertiles of each protein. Blue, tertile 1 (T1, lowest protein levels); gray, tertile 2 (T2); red, tertile 3 (T3, highest protein levels). (c and f) Forest plots of per tertile analyses for S100A12 in *SAHLSIS* and *SAHLSIS2*, respectively, with the lowest tertile (T1) as reference.

Results for proteins that showed nominal associations to recurrent stroke in univariable or multivariable Cox regression analyses in *SAHLSIS*, as well as results on hsCRP and IL-6 for comparison, are shown in Figure 3(a). Results for all 66 proteins are available in the Online Supplement (Supplemental Table S4). Elevated S100A12 was nominally associated with an increased risk of stroke recurrence in both univariable (HR [95% CI]: 1.28 [1.09–1.51], *p* = 0.002 per NPX unit) and multivariable (adjusted HR [95% CI]: 1.22 [1.02–1.46], *p* = 0.03) analyses, and elevated plasma levels of CCL23 was nominally associated with a decreased risk of stroke recurrence (adjusted HR [95% CI]: 0.67 [0.47–0.97], *p* = 0.03 per NPX unit).

S100A12 also demonstrated a difference in survival curves between protein tertiles with highest cumulative incidence of stroke recurrence in the highest tertile (standard log-rank *p* = 0.017; Figure 3(b)). On per tertile Cox regression analysis, S100A12 showed a dose-response association with the highest risk for stroke recurrence in the highest tertile (adjusted HR 1.51 [1.00–2.29], T3 vs T1; Figure 3(c)).

The associations for S100A12 and recurrent stroke were replicated in *SAHLSIS2* (univariable HR [95% CI]: 1.36 [1.13–1.63], *p* = 0.001 per NPX unit; adjusted HR: 1.35 [1.10–1.66], *p* = 0.004; Figure 3(d)). The highest S100A12 tertile had the greatest cumulative incident of recurrent stroke (Figure 3(e)). After adjustment for vascular risk factors and statin use, patients in the top tertile of S100A12 had a two-fold increased risk of stroke recurrence compared with patients in the bottom tertile (adjusted HR 1.96 [0.99–3.89], T3 vs T1; Figure 3(f)).

### Exploratory subtype-specific stratified analysis for recurrent MACE

**Figure 4.**
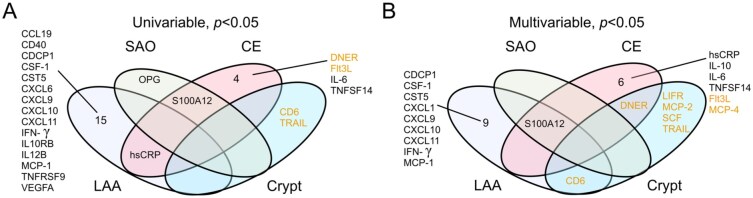
Venn diagram showing unique and shared protein associations with recurrent MACE at *p* < 0.05 across the four main etiological ischemic stroke subtypes. (a) Univariable Cox regression; (b) Multivariable Cox regression models adjusted for age, sex, day of blood draw, hypertension, diabetes mellitus, previous coronary artery disease, and statin treatment.

Finally, we explored whether there were etiologic subtype-specific associations between each of the 66 proteins and MACE in *SAHLSIS* for each of the four main subtypes (LAA, SAO, CE and cryptogenic stroke) of ischemic stroke. Baseline characteristics for each subtype are summarized in Supplemental Table S5. Results from the univariable and multivariable Cox regressions are summarized in Figure 4 and details from both analyses are available in the Online Supplement (Supplemental Table S6). LAA had the highest rate of MACE (67%) during the median 14.7 year follow-up, followed by CE (53%), SAO (50%), and cryptogenic (28%) stroke. LAA and CE stroke had the shortest median time to MACE (1861 and 1891 days, respectively), followed by cryptogenic stroke (2492 days) and SAO stroke (3006 days). The subtype with the most proteins associated with MACE at *p* < 0.05 was LAA (*n* = 16 proteins in univariable analyses including hsCRP; and *n* = 11 in multivariable analyses including S100A12), and for most proteins, elevated levels were associated with increased risk of MACE. This contrasts with the other subtypes (Figure 4). In adjusted analyses for CE stroke, elevated levels of 5 proteins (hsCRP, IL-6, IL-10, S100A12, and TNFSF14) were associated with increased risk of MACE, whereas elevated levels of 3 proteins (DNER, Flt3L, and MCP-4) were associated with decreased risk. For cryptogenic stroke, elevated levels of 6 proteins were associated with decreased risk of MACE after adjustment (CD-6, DNER, LIF-R, MCP-2, SCF, and TRAIL). In SAO stroke, elevated S100A12 was associated with increased risk of MACE.

## Discussion

Despite modern guideline-based secondary prevention, ischemic stroke survivors face a high risk of vascular recurrence. There is a need for improved risk prediction to identify patients at greatest risk of vascular recurrence, and blood-based inflammatory biomarkers hold promise.^9^ Here, using a proteomic profiling approach targeting the inflammatory pathway, we identified S100A12 as a novel blood biomarker for vascular recurrence following ischemic stroke in a cohort of adult patients with ischemic stroke before 70 years of age. The associations to recurrent MACE and stroke, including dose-response associations, were replicated in an independent older ischemic stroke cohort, suggesting that S100A12 is a novel and robust blood biomarker of vascular recurrence across age groups.

S100A12 [alias EN-RAGE or calgranulin C] is a member of the S100 family of calcium-binding proteins constitutively expressed by neutrophils and inducible in macrophages.^20^ When secreted extracellularly, S100A12 acts as an “alarmin” or damage associated molecular pattern (DAMP) and is a ligand for pattern recognition receptors such as RAGE (receptor for advanced glycation end products) and TLR-4 (toll-like receptor 4).^21,22^ Upon receptor binding, it induces the production of pro-inflammatory cytokines, such as IL-1β, IL-6, and TNF-α, via activation of the MAP-kinase and NF-κB intracellular signaling pathways.^21,23,24^ S100A12 has been implicated in the pathogenesis of atherosclerosis,^23,25^ and contributes to vascular inflammation, vascular calcification, and vascular oxidative stress.^26^ In clinical studies, elevated circulating levels of S100A12 have been associated with increased risk of incident coronary heart disease,^24,27^ heart failure,^28,29^ and more extensive coronary atherosclerosis in patients with CAD, diabetes mellitus and chronic kidney disease.^30–35^ In the context of stroke, we have found that plasma levels of S100A12 are elevated in ischemic stroke patients compared to healthy controls, not only during the acute-phase, but also during the convalescent-phase (i.e. at 3-month follow-up), and remain elevated even 7 years after the index stroke.^18^ Furthermore, we and others have found that high acute-phase plasma levels of S100A12 are associated with unfavorable functional outcome after ischemic stroke.^25,36,37^ With regards to vascular recurrence, the present findings are in line with one study on 524 patients with an acute coronary syndrome which showed that increased acute phase plasma levels of S100A12 were associated with increased risk of MACE independently of vascular risk factors.^38^ Taken together, the results from our study thus supports previous findings on the role of S100A12 as an important inflammatory mediator in cardiovascular disease and show for the first time its potential value as a prognostic biomarker also for vascular recurrence after ischemic stroke.

The present data also suggest that S100A12 inhibition might be a therapeutic target for secondary prevention after ischemic stroke. In recent years, the concept of targeting pro-inflammatory mechanisms for secondary prevention in cardiovascular diseases has gained traction. For instance, a recent meta-analysis of randomized controlled trials on the anti-inflammatory drug colchicine in patients with CAD or stroke (including the CONVINCE and CHANCE-3 studies on stroke) showed that colchicine provides a 12% relative reduction in the rate of MACE.^39^ Inhibiting the S100/calgranulin-mediated activation of RAGE has been suggested as a therapeutic target for atherosclerosis^25^ and may therefore represent an alternative target for secondary prevention. In support of this theory, the immune-modulatory compound ABR-215757, a quinoline-3-carboxamide, has been demonstrated to bind S100A12 and attenuate S100A12-mediated features of atherosclerosis in experimental models.^40^ In this context it is of interest to note that two antiallergic drugs, Olopatadine and Amlexanox, have been shown to bind to S100A12^41^ and suppress S100A12 induced monocyte migration.^42^ Although their exact pharmacological mechanisms of action remain unknown, one might speculate, that these two and/or other drugs might be candidates for drug repurposing for secondary prevention after ischemic stroke and other vascular events.

We also performed exploratory analyses of associations between the inflammatory proteins and recurrent MACE separately in the four main etiological subtypes of ischemic stroke in *SAHLSIS*. Given the relatively low number of events in each subtype, we emphasize that these analyses are clearly only exploratory and thus the results should be interpreted with caution. Nonetheless, there were several striking differences worth noting that are in line with the literature and thus biologically plausible. The subtype of LAA stroke had the largest number of proteins that were associated with recurrent MACE (*n* = 16 in univariable analyses including hsCRP; and *n* = 11 in multivariable analyses including S100A12). For most proteins, elevated levels increased the risk of MACE, and many of the proteins are known to participate in various aspects of atherosclerotic disease progression (e.g. MCP-1,^43^ CSF-1,^44^ CXCL1, CXCL9, CXCL10, CXCL11, and interferon gamma).^45^ In line with these results, Mendelian Randomization studies have shown higher genetically predicted MCP-1 and CSF-1 to be associated with increased risk of any ischemic stroke and LAA stroke.^46,47^ In CE stroke, increased levels of hsCRP, IL-6, IL-10, S100A12 and TNFSF14 were independently associated with an increased risk of MACE while DNER, Flt3L and MCP-4 were associated with a decreased risk. For hsCRP and IL-6 this is in line with previous studies.^9–11^ While studies on TNFSF14 and vascular recurrence after ischemic stroke outcomes are lacking, increased TNFSF14 predicts MACE in patients with stable coronary artery disease,^48^ which is directionally concordant to the association observed here. Flt3L is a cytokine that stimulates the proliferation of primitive hematopoietic progenitor cells and is involved in the development of dendritic cells. Low Flt3L levels and impaired dendritic cell differentiation have been reported in patients with coronary artery disease compared to controls,^49^ which is in line with the direction of the association in CE stroke observed here. Of interest, the findings for CE stroke and especially LAA stroke contrast with results for cryptogenic stroke (defined here as unknown cause in cases with complete evaluation; and does not include cases with more than one possible cause), where elevated levels of six proteins, including LIFR and SCF, were associated with a decreased risk of recurrent MACE. In Mendelian Randomization analyses, genetically determined levels of LIFR were associated with reduced risk of stroke and with lower risk of both angina pectoris and atrial fibrillation.^50^ SCF plays a role in vascular repair and lower circulating levels of SCF have been associated with an increased risk of stroke,^51^ cardiovascular mortality^51^ and incident coronary events,^52^ in line with our results. Taken together, although many of these subtype-specific associations seem biologically plausible, associations between these proteins and recurrent MACE need of course to be taken with caution due to the small sample size and investigations of subtype-specific association of inflammation-related plasma proteins in larger ischemic stroke cohorts are thus warranted.

CD6 was nominally associated with a decreased risk of recurrent MACE in the multivariable analysis in all ischemic stroke in the younger *SAHLSIS* cohort but did not replicate in the older *SAHLSIS2* cohort. Speculatively, this may be due to the different subtype distribution in the two cohorts, as the association was partially driven by cryptogenic stroke which is more common in *SAHLSIS* (26%) compared to *SAHLSIS2* (16%).

Strengths of this study include the inclusion of clinically well-characterized study participants as well as standardized pre-analytical conditions for measuring plasma levels of a large number of proteins by a multiplex method with high sensitivity and specificity. For discovery, we focused on young and middle-age ischemic stroke cases, meaning that confounding by comorbidities is limited and that the survival rate is high which enabled us to follow the study participants for many years. This long-term follow-up had minimal missingness due to the very high coverage in the Swedish national registers and was of high quality, as we validated the events recorded in these registers by review of medical records. Furthermore, we replicated our main finding for S100A12 in an independent ischemic cohort of all ages, which provides some evidence for generalizability across age groups.

We acknowledge that there are also important study limitations to consider. Firstly, as our sample size for discovery is modest there may be other proteins that are associated with vascular recurrence that were not detected here. Secondly, the day of blood draw was not standardized. However, the day of blood draw was used as a covariate in the multivariable analyses, and we have previously shown that it displays only a weak to no correlation with the plasma levels of the proteins investigated here.^18^ Thirdly, there is a temporal difference between the two cohorts, and of note the study participants in the discovery cohort (i.e. *SAHLSIS*) were recruited in a time era when acute revascularization therapies were not yet part of standard care. Thus, we recognize that if there are proteins that are associated with vascular recurrence specifically in patients undergoing revascularization therapy they were not detected here. We also had a slight discrepancy in the definition of the coronary events between the two cohorts in that codes for percutaneous interventions or coronary bypass grafting were used to define events in *SAHLSIS* only. However, this was deemed acceptable as these interventions made up only a negligible portion of the total amount of coronary events *SAHLSIS*. Furthermore, as both cohorts were recruited from the same area of Sweden, the results may not be generalizable to other populations. Moreover, as indicated above, subtype-stratified analyses clearly require further study in larger cohorts. Here it is also noted that the low number of events per determined stroke subtype in *SAHLSIS2* prohibited its use for validation of the subtype-stratified results. This was due both to the larger proportion of cases with an undetermined stroke subtype in this older cohort and the shorter follow-up time. Finally, although we performed multivariable analyses adjusted for conventional cardiovascular risk factors and statin use, we cannot entirely exclude the possibility of residual confounding. In the future, it would therefore also be of interest to explore other possible between-group differences and perform additional subgroup analyses (e.g. based on prior CAD or diabetes mellitus) in larger cohorts.

In conclusion, this study identifies S100A12 as a novel blood biomarker of vascular recurrence after ischemic stroke. The findings indicate that S100A12 might be a potential therapeutic target for secondary prevention warranting further study. We also identified etiologic subtype-specific associations for several proteins with recurrent MACE, warranting further exploration in larger cohorts.

## Supplementary Material

ds-eso_23969873251384439

## Data availability

Anonymized data will be shared upon reasonable request, provided data transfer agrees with EU legislation on the general data protection regulation and with decisions by the Ethical Review Board of Sweden and the University of Gothenburg, the latter which should be regulated in a data transfer agreement.
