# Supplementary material for: Elevated acute-phase plasma levels of S100A12 [EN-RAGE] are associated with vascular recurrence after ischemic stroke
Source: Eur Stroke J. 2026 Jan 1;11(1):23969873251384439. doi: 10.1093/esj/23969873251384439 (PMC12866258; doi:10.1093/esj/23969873251384439)
Supplement: ds-eso_23969873251384439 [file ds-eso_23969873251384439.zip › sj-pdf-1-eso-10.1177_23969873251384439.pdf]

Supplementary Material

Elevated plasma S100A12 is associated with increased risk of vascular recurrence after ischemic stroke

Björn Granelli[1]*, Annelie Angerfors[1]*, Sofia Furutjäll[1], Hanh Nguyen Larsson[1], Cecilia Brännmark[2], Björn Andersson[3], Tara M Stanne[1,4]‡†, Christina Jern[1,4]‡

* These authors equally contributed to this work.
‡ These authors jointly supervised this work.

Affiliations:
[1]Institute of Biomedicine, Department of Laboratory Medicine, Sahlgrenska Academy, University of Gothenburg, Gothenburg, Sweden
[2]Department of Medicine, Geriatrics and Emergency Medicine, Sahlgrenska University Hospital/Östra Sjukhuset, Gothenburg, Sweden
[3]Bioinformatics and Data Center, Core Facilities, Sahlgrenska Academy, University of Gothenburg, Gothenburg, Sweden
[4]Region Västra Götaland, Sahlgrenska University Hospital, Department of Clinical Genetics and Genomics, Gothenburg, Sweden

Short title: S100A12 and risk of vascular recurrence after ischemic stroke

†**Corresponding author**: Tara M Stanne, PhD, Associate Professor
Institute of Biomedicine, the Sahlgrenska Academy, University of Gothenburg, Box 440, SE-405 30 Gothenburg, Sweden.
E-mail: tara.stanne@gu.se

Supplementary Tables

Table S1. Hazard ratios (HR) and 95% confidence intervals (CI) for MACE per doubling of protein levels from univariable and multivariable Cox regressions.

Protein	MACE (Univariable)			MACE (Multivariable)		
	HR (95% CI)	P-value	FDR	HR (95% CI)	P-value	FDR
4E-BP1	1.02 (0.93-1.13)	0.63	0.87	1.02 (0.92-1.14)	0.67	0.91
ADA	0.96 (0.74-1.24)	0.75	0.87	1.00 (0.75-1.32)	0.98	0.98
AXIN1	1.04 (0.94-1.15)	0.42	0.75	1.05 (0.95-1.16)	0.36	0.90
CASP-8	1.03 (0.84-1.26)	0.80	0.89	1.03 (0.83-1.27)	0.80	0.92
CCL11	1.29 (1.02-1.62)	0.03	0.22	1.06 (0.82-1.37)	0.67	0.91
CCL19	1.13 (1.00-1.29)	0.06	0.29	1.07 (0.93-1.23)	0.37	0.90
CCL20	0.99 (0.88-1.11)	0.85	0.90	0.98 (0.86-1.12)	0.77	0.92
CCL23	0.98 (0.76-1.27)	0.88	0.92	0.94 (0.70-1.26)	0.68	0.91
CCL25	1.29 (1.07-1.55)	0.01	0.14	1.18 (0.96-1.43)	0.11	0.87
CCL28	0.98 (0.76-1.28)	0.90	0.93	0.85 (0.63-1.16)	0.31	0.90
CCL3	1.08 (0.86-1.36)	0.51	0.85	0.93 (0.71-1.21)	0.57	0.91
CCL4	0.87 (0.71-1.07)	0.18	0.54	0.88 (0.71-1.09)	0.25	0.90
CD244	0.84 (0.6-1.17)	0.31	0.65	0.85 (0.60-1.21)	0.37	0.90
CD40	1.27 (0.98-1.66)	0.07	0.29	1.11 (0.84-1.48)	0.45	0.90
CD5	0.93 (0.69-1.26)	0.63	0.87	0.84 (0.60-1.17)	0.31	0.90
CD6	0.80 (0.63-1.01)	0.06	0.29	0.77 (0.60-0.99)	0.04	0.87
CDCP1	1.25 (1.03-1.51)	0.02	0.21	0.94 (0.75-1.18)	0.60	0.91
**hsCRP**	1.10 (1.04-1.18)	**2e-03**	**0.04**	1.05 (0.97-1.12)	0.22	0.90
CSF-1	1.20 (0.84-1.71)	0.32	0.65	1.10 (0.75-1.61)	0.63	0.91
CST5	1.26 (0.98-1.63)	0.08	0.29	1.10 (0.84-1.45)	0.48	0.90
CX3CL1	0.94 (0.72-1.24)	0.66	0.87	0.79 (0.60-1.06)	0.12	0.87
CXCL1	0.97 (0.87-1.07)	0.51	0.85	0.99 (0.89-1.11)	0.88	0.93
CXCL10	1.04 (0.91-1.18)	0.60	0.87	0.96 (0.83-1.11)	0.57	0.91
CXCL11	1.04 (0.92-1.17)	0.57	0.85	0.99 (0.87-1.13)	0.89	0.93
CXCL5	0.98 (0.91-1.06)	0.63	0.87	0.99 (0.91-1.06)	0.70	0.91
CXCL6	1.00 (0.85-1.17)	0.97	0.97	0.98 (0.83-1.17)	0.85	0.93
CXCL9	1.03 (0.90-1.16)	0.70	0.87	0.94 (0.81-1.09)	0.39	0.90
DNER	0.62 (0.42-0.91)	0.01	0.16	0.75 (0.50-1.14)	0.18	0.90
FGF-19	1.06 (0.93-1.21)	0.39	0.74	1.10 (0.96-1.26)	0.19	0.90
FGF-21	1.05 (0.96-1.16)	0.30	0.65	1.01 (0.90-1.12)	0.93	0.94
FGF-23	1.25 (1.04-1.51)	0.02	0.17	1.18 (0.97-1.44)	0.10	0.87
Flt3L	1.00 (0.78-1.27)	0.97	0.97	0.89 (0.69-1.15)	0.38	0.90
hGDNF	1.09 (0.82-1.44)	0.56	0.85	0.94 (0.69-1.29)	0.71	0.91
HGF	1.02 (0.87-1.18)	0.83	0.90	0.98 (0.82-1.16)	0.81	0.92

…

Table S1, continued. Hazard ratios (HR) and 95% confidence intervals (CI) for MACE per doubling of protein levels from univariable and multivariable Cox regressions.

Protein	MACE (Univariable)			MACE (Multivariable)		
	HR (95% CI)	P-value	FDR	HR (95% CI)	P-value	FDR
IFNgamma	0.93 (0.74-1.17)	0.55	0.85	0.98 (0.77-1.25)	0.86	0.93
IL-10	1.06 (0.90-1.26)	0.49	0.85	1.07 (0.90-1.27)	0.45	0.90
IL-10RB	1.23 (0.93-1.63)	0.15	0.50	1.03 (0.77-1.39)	0.83	0.93
IL-12B	1.11 (0.93-1.32)	0.26	0.64	1.03 (0.85-1.25)	0.76	0.92
IL-18	1.14 (0.93-1.39)	0.20	0.55	1.09 (0.88-1.35)	0.42	0.90
IL-18R1	0.96 (0.73-1.26)	0.76	0.87	0.85 (0.63-1.15)	0.30	0.90
IL-6	1.10 (0.99-1.21)	0.07	0.29	1.06 (0.95-1.19)	0.28	0.90
IL-7	0.97 (0.82-1.15)	0.70	0.87	0.97 (0.81-1.15)	0.71	0.91
IL-8	1.11 (0.96-1.29)	0.17	0.54	1.01 (0.85-1.20)	0.90	0.93
LAP TGF-beta-1	0.88 (0.69-1.12)	0.29	0.65	0.81 (0.62-1.04)	0.10	0.87
LIF-R	0.81 (0.57-1.16)	0.25	0.64	0.68 (0.46-0.99)	0.05	0.87
MCP-1	1.14 (0.90-1.46)	0.28	0.65	0.91 (0.70-1.18)	0.48	0.90
MCP-2	1.03 (0.86-1.25)	0.73	0.87	0.91 (0.75-1.11)	0.36	0.90
**MCP-3**	1.32 (1.11-1.58)	**2e-03**	**0.04**	1.09 (0.89-1.34)	0.41	0.90
MCP-4	1.03 (0.85-1.25)	0.75	0.87	0.84 (0.69-1.03)	0.10	0.87
MMP-10	1.03 (0.88-1.21)	0.69	0.87	1.06 (0.89-1.26)	0.52	0.90
NT-3	1.12 (0.89-1.40)	0.35	0.68	1.04 (0.84-1.28)	0.73	0.92
OPG	1.46 (1.08-1.96)	0.01	0.16	1.09 (0.78-1.52)	0.62	0.91
OSM	0.95 (0.85-1.07)	0.41	0.75	0.96 (0.84-1.09)	0.51	0.90
**S100A12**	1.36 (1.20-1.53)	**9e-07**	**6e-05**	**1.27 (1.10-1.45)**	**8e-04**	0.052
SCF	0.82 (0.66-1.02)	0.07	0.29	0.81 (0.64-1.03)	0.08	0.87
SIRT2	1.01 (0.93-1.11)	0.75	0.87	1.03 (0.94-1.13)	0.57	0.91
SLAMF1	1.16 (0.93-1.46)	0.20	0.55	0.97 (0.75-1.25)	0.79	0.92
STAMBP	1.04 (0.92-1.17)	0.54	0.85	1.05 (0.92-1.19)	0.48	0.90
TNFB	1.04 (0.83-1.30)	0.76	0.87	1.13 (0.89-1.43)	0.32	0.90
TNFRSF9	1.29 (1.01-1.65)	0.04	0.26	1.15 (0.88-1.50)	0.32	0.90
TNFSF14	1.12 (0.89-1.41)	0.33	0.66	1.06 (0.82-1.38)	0.64	0.91
TRAIL	0.81 (0.61-1.08)	0.15	0.50	0.84 (0.61-1.15)	0.28	0.90
TRANCE	0.91 (0.76-1.08)	0.26	0.64	0.90 (0.75-1.08)	0.27	0.90
TWEAK	0.80 (0.62-1.04)	0.09	0.33	0.82 (0.62-1.09)	0.17	0.90
uPA	0.96 (0.68-1.35)	0.81	0.89	0.92 (0.65-1.29)	0.62	0.91
VEGFA	1.34 (1.02-1.76)	0.03	0.22	1.11 (0.82-1.51)	0.49	0.90

Grey shaded, nominally significant in one or more analyses; Bold, FDR significant; Multivariable: adjusted for age, sex, day of blood draw, hypertension, diabetes mellitus, previous CAD, and statin treatment at discharge

Table S2. Hazard ratios (HR) and 95% confidence intervals (CI) for MACE per doubling of protein levels from univariable and multivariable Cox regressions in sensitivity analyses including only MACE within 6-year follow-up.

	MACE within 2184 days (Univariable)			MACE within 2184 days (Multivariable)		
**Protein**	**HR (95% CI)**	**P-value**	**FDR**	**HR (95% CI)**	**P-value**	**FDR**
CCL11	1.38 (1.00-1.90)	0.048	0.09	1.16 (0.81-1.66)	0.43	0.93
CCL25	1.15 (0.88-1.49)	0.32	0.32	1.03 (0.77-1.37)	0.86	0.98
CD6	0.84 (0.60-1.17)	0.29	0.32	0.80 (0.56-1.14)	0.22	0.93
CDCP1	1.28 (0.98-1.68)	0.07	0.10	1.02 (0.74-1.41)	0.89	0.98
**hsCRP**	**1.16 (1.06-1.26)**	**1e-03**	**8e-03**	1.09 (0.98-1.20)	0.10	0.93
DNER	0.65 (0.38-1.12)	0.12	0.15	0.98 (0.54-1.78)	0.96	0.99
**FGF23**	**1.41 (1.12-1.77)**	**4e-03**	**0.02**	1.27 (0.99-1.63)	0.06	0.93
IL-6	1.16 (1.02-1.32)	0.03	0.06	1.12 (0.96-1.31)	0.14	0.93
**MCP-3**	**1.36 (1.07-1.72)**	**0.01**	**0.04**	1.09 (0.81-1.46)	0.56	0.93
OPG	1.49 (0.97-2.28)	0.07	0.10	1.12 (0.70-1.80)	0.64	0.93
**S100A12**	**1.45 (1.23-1.70)**	**7e-06**	**9e-05**	1.34 (1.11-1.61)	2e-03	0.17
TNFRSF9	1.49 (1.06-2.09)	0.02	0.052	1.26 (0.87-1.83)	0.22	0.93
VEGF-A	1.35 (0.92-1.97)	0.13	0.15	1.01 (0.65-1.57)	0.96	0.99

Grey shaded, nominally significant in one or more analyses; Bold, FDR significant; Multivariable: adjusted for age, sex, day of blood draw, hypertension, diabetes mellitus, previous CAD, and statin treatment at discharge

Table S3. Hazard ratios (HR) and 95% confidence intervals (CI) for MACE per doubling of protein levels from univariable and multivariable Cox regressions in sensitivity analyses including only first-ever stroke.

	MACE First stroke only (Univariable)			MACE First stroke only (Multivariable)		
**Protein**	**HR (95% CI)**	**P-value**	**FDR**	**HR (95% CI)**	**P-value**	**FDR**
CCL11	1.24 (0.94-1.64)	0.14	0.16	1.00 (0.73-1.36)	0.98	0.98
CCL25	1.35 (1.08-1.69)	0.01	0.04	1.20 (0.95-1.52)	0.12	0.92
CD6	0.83 (0.63-1.09)	0.18	0.20	0.84 (0.63-1.12)	0.24	0.92
CDCP1	1.22 (0.97-1.53)	0.09	0.16	0.90 (0.68-1.18)	0.43	0.92
**hsCRP**	**1.11 (1.03-1.19)**	**4e-03**	**0.03**	1.05 (0.97-1.14)	0.26	0.92
DNER	0.63 (0.41-0.97)	0.04	0.09	0.76 (0.47-1.22)	0.25	0.92
FGF23	1.20 (0.95-1.51)	0.13	0.16	1.19 (0.94-1.51)	0.16	0.92
IL6	1.09 (0.98-1.22)	0.12	0.16	1.06 (0.94-1.20)	0.36	0.92
MCP3	1.28 (1.04-1.57)	0.02	0.06	1.05 (0.83-1.34)	0.67	0.92
**S100A12**	**1.36 (1.18-1.56)**	**2e-05**	**2e-04**	1.29 (1.11-1.51)	1e-03	0.07
OPG	1.44 (1.01-2.05)	0.04	0.09	1.04 (0.70-1.55)	0.83	0.95
TNFRSF9	1.17 (0.87-1.58)	0.30	0.30	1.08 (0.78-1.49)	0.64	0.92

Grey shaded, nominally significant in one or more analyses; Bold, FDR significant; Multivariable: adjusted for age, sex, day of blood draw, hypertension, diabetes mellitus, previous CAD, and statin treatment at discharge

Table S4. Hazard ratios (HR) and 95% confidence intervals (CI) for recurrent stroke per doubling of protein levels from univariable and multivariable Cox regressions.

Protein	Recurrent stroke (Univariable)			Recurrent stroke (Multivariable)		
	HR (95% CI)	P-value	FDR	HR (95% CI)	P-value	FDR
4E-BP1	0.98 (0.86-1.11)	0.71	0.85	0.96 (0.84-1.10)	0.59	0.83
ADA	0.80 (0.57-1.14)	0.21	0.84	0.87 (0.61-1.26)	0.47	0.80
AXIN1	0.98 (0.86-1.12)	0.80	0.94	1.00 (0.87-1.14)	0.98	0.98
CASP-8	0.83 (0.62-1.10)	0.19	0.84	0.84 (0.63-1.13)	0.26	0.80
CCL11	1.18 (0.87-1.60)	0.29	0.84	1.08 (0.77-1.52)	0.64	0.83
CCL19	1.08 (0.91-1.28)	0.37	0.84	1.05 (0.88-1.26)	0.58	0.83
CCL20	0.93 (0.79-1.09)	0.36	0.84	0.94 (0.79-1.11)	0.45	0.80
CCL23	0.72 (0.51-1.01)	0.06	0.84	0.67 (0.46-0.97)	0.03	0.80
CCL25	1.11 (0.87-1.42)	0.39	0.84	1.05 (0.81-1.35)	0.73	0.88
CCL28	1.08 (0.77-1.50)	0.66	0.85	1.01 (0.70-1.47)	0.94	0.98
CCL3	0.90 (0.66-1.23)	0.51	0.85	0.83 (0.59-1.17)	0.29	0.80
CCL4	0.86 (0.65-1.12)	0.26	0.84	0.88 (0.66-1.17)	0.39	0.80
CD244	0.90 (0.59-1.38)	0.63	0.85	1.01 (0.65-1.57)	0.96	0.98
CD40	0.93 (0.65-1.34)	0.70	0.85	0.88 (0.61-1.29)	0.52	0.80
CD5	0.71 (0.48-1.07)	0.10	0.84	0.69 (0.45-1.06)	0.09	0.80
CD6	0.86 (0.64-1.17)	0.34	0.84	0.88 (0.64-1.20)	0.41	0.80
CDCP1	1.03 (0.79-1.33)	0.84	0.95	0.84 (0.63-1.13)	0.24	0.80
hsCRP	1.06 (0.98-1.15)	0.15	0.84	1.01 (0.92-1.10)	0.89	0.98
CSF-1	0.87 (0.55-1.39)	0.57	0.85	0.83 (0.50-1.35)	0.45	0.80
CST5	0.92 (0.65-1.28)	0.61	0.85	0.86 (0.60-1.23)	0.40	0.80
CX3CL1	0.79 (0.55-1.14)	0.21	0.84	0.75 (0.51-1.09)	0.13	0.80
CXCL1	0.90 (0.79-1.03)	0.12	0.84	0.92 (0.80-1.06)	0.25	0.80
CXCL10	1.00 (0.84-1.18)	0.96	0.99	0.96 (0.81-1.15)	0.67	0.83
CXCL11	0.93 (0.78-1.09)	0.36	0.84	0.93 (0.78-1.10)	0.39	0.80
CXCL5	0.94 (0.86-1.03)	0.18	0.84	0.94 (0.85-1.03)	0.19	0.80
CXCL6	0.90 (0.72-1.11)	0.33	0.84	0.92 (0.74-1.15)	0.46	0.80
CXCL9	0.99 (0.84-1.17)	0.92	0.97	0.93 (0.78-1.12)	0.47	0.80
DNER	0.74 (0.45-1.21)	0.23	0.84	0.92 (0.54-1.58)	0.77	0.89
FGF-19	1.12 (0.95-1.33)	0.19	0.84	1.15 (0.97-1.37)	0.11	0.80
FGF-21	1.05 (0.93-1.19)	0.44	0.85	0.98 (0.85-1.12)	0.73	0.88
FGF-23	1.10 (0.85-1.42)	0.48	0.85	1.09 (0.83-1.42)	0.54	0.80
Flt3L	0.87 (0.64-1.18)	0.37	0.84	0.82 (0.59-1.14)	0.24	0.80
hGDNF	0.91 (0.63-1.31)	0.61	0.85	0.83 (0.56-1.24)	0.37	0.80
HGF	1.01 (0.83-1.23)	0.89	0.95	0.99 (0.79-1.24)	0.95	0.98

…

Table S4, continued. Hazard ratios (HR) and 95% confidence intervals (CI) for recurrent stroke per doubling of protein levels from univariable and multivariable Cox regressions.

Protein	Recurrent stroke (Univariable)			Recurrent stroke (Multivariable)		
	HR (95% CI)	P-value	FDR	HR (95% CI)	P-value	FDR
IFNgamma	0.83 (0.60-1.15)	0.26	0.84	0.87 (0.63-1.21)	0.42	0.80
IL-10	1.00 (0.80-1.26)	0.99	0.99	1.00 (0.80-1.26)	0.98	0.98
IL-10RB	0.92 (0.64-1.34)	0.68	0.85	0.86 (0.59-1.26)	0.45	0.80
IL-12B	1.06 (0.84-1.33)	0.63	0.85	1.08 (0.85-1.38)	0.52	0.80
IL-18	0.93 (0.71-1.21)	0.60	0.85	0.91 (0.69-1.21)	0.53	0.80
IL-18R1	0.82 (0.57-1.17)	0.27	0.84	0.74 (0.50-1.09)	0.13	0.80
IL-6	1.07 (0.94-1.22)	0.30	0.84	1.02 (0.88-1.18)	0.79	0.89
IL-7	0.80 (0.64-1.01)	0.06	0.84	0.83 (0.66-1.06)	0.13	0.80
IL-8	0.98 (0.80-1.21)	0.86	0.95	0.95 (0.75-1.19)	0.63	0.83
LAP TGF-beta-1	0.73 (0.52-1.01)	0.053	0.84	0.72 (0.51-1.01)	0.06	0.80
LIF-R	0.78 (0.49-1.25)	0.30	0.84	0.71 (0.44-1.17)	0.18	0.80
MCP-1	0.93 (0.67-1.29)	0.66	0.85	0.80 (0.57-1.13)	0.20	0.80
MCP-2	0.93 (0.73-1.19)	0.59	0.85	0.86 (0.67-1.11)	0.25	0.80
MCP-3	1.22 (0.97-1.54)	0.09	0.84	1.10 (0.84-1.42)	0.49	0.80
MCP-4	1.02 (0.80-1.30)	0.88	0.95	0.92 (0.72-1.19)	0.54	0.80
MMP-10	0.92 (0.74-1.15)	0.46	0.85	0.93 (0.73-1.17)	0.53	0.80
NT-3	1.09 (0.81-1.48)	0.56	0.85	1.06 (0.80-1.42)	0.67	0.83
OPG	1.17 (0.79-1.74)	0.43	0.85	0.88 (0.57-1.35)	0.55	0.80
OSM	0.91 (0.78-1.06)	0.23	0.84	0.88 (0.74-1.03)	0.11	0.80
S100A12	1.28 (1.09-1.51)	2e-03	0.16	1.21 (1.01-1.45)	0.04	0.80
SCF	0.97 (0.72-1.31)	0.86	0.95	1.02 (0.74-1.41)	0.90	0.98
SIRT2	0.95 (0.85-1.07)	0.40	0.84	0.96 (0.85-1.09)	0.54	0.80
SLAMF1	1.13 (0.84-1.52)	0.41	0.84	1.08 (0.79-1.49)	0.62	0.83
STAMBP	0.96 (0.83-1.13)	0.65	0.85	0.98 (0.83-1.15)	0.77	0.89
TNFB	1.00 (0.75-1.34)	0.98	0.99	1.13 (0.84-1.52)	0.42	0.80
TNFRSF9	1.12 (0.81-1.55)	0.50	0.85	1.13 (0.80-1.58)	0.49	0.80
TNFSF14	0.94 (0.69-1.28)	0.69	0.85	0.90 (0.63-1.27)	0.53	0.80
TRAIL	0.79 (0.54-1.15)	0.21	0.84	0.83 (0.56-1.25)	0.37	0.80
TRANCE	1.02 (0.81-1.28)	0.88	0.95	1.06 (0.84-1.35)	0.63	0.83
TWEAK	0.92 (0.67-1.28)	0.62	0.85	1.02 (0.72-1.45)	0.91	0.98
uPA	0.72 (0.46-1.13)	0.15	0.84	0.80 (0.52-1.25)	0.34	0.80
VEGFA	0.90 (0.61-1.31)	0.57	0.85	0.75 (0.50-1.13)	0.17	0.80

Grey shaded, nominally significant in one or more analyses; Multivariable: adjusted for age, sex, day of blood draw, hypertension, diabetes mellitus, previous CAD, and statin treatment at discharge

Table S5. Baseline characteristics for each of the four main subtypes of ischemic stroke in *SAHLSIS.* Data are shown as median and interquartile range (IQR), or number (n) and percentage.

	LAA		SAO		CE		Cryptogenic	
	No event	MACE	No event	MACE	No event	MACE	No event	MACE
n (%)	24 (33%)	49 (67%)	62 (50%)	61 (50%)	46 (47%)	52 (53%)	116 (72%)	45 (28%)
Age in years, median [IQR]	59 [52-60]	62 [58-67]	58 [52-62]	62 [57-66]	59 [54-64]	63 [52-67]	54 [45-60]	61 [56-65]
Sex, Male (%)	18 (75%)	36 (73%)	38 (61%)	38 (62%)	33 (72%)	33 (63%)	65 (56%)	30 (67%)
Hypertension, n (%)	11 (46%)	33 (72%)	43 (69%)	46 (75%)	21 (46%)	29 (58%)	54 (47%)	33 (73%)
Diabetes mellitus, n (%)	3 (13%)	22 (45%)	12 (19%)	14 (23%)	5 (11%)	14 (27%)	14 (12%)	9 (20%)
Preexisting CAD, n (%)	0 (0%)	12 (26%)	3 (5%)	5 (8%)	9 (22%)	18 (38%)	6 (6%)	4 (10%)
Statin treatment, n (%)	12 (52%)	25 (56%)	19 (33%)	20 (33%)	13 (32%)	18 (38%)	35 (33%)	11 (27%)
Time (days) to follow up, median [IQR]	7028 [5206-7301]	1861 [352-3857]	7003 [6591-7463]	3006 [1508-4912]	6742 [5315-7157]	1891 [962-3738]	7236 [6700-7565]	2492 [887-4561]

Table S6. Hazard ratios (HR) and 95% confidence intervals (CI) for MACE stratified by etiologic ischemic stroke subtype per doubling of protein levels from univariable and multivariable Cox regressions.

	**Univariable**							
	**LAA**		**CE**		**SAO**		**Cryptogenic**	
**Protein**	**HR (95% CI)**	**p-value**	**HR (95% CI)**	**p-value**	**HR (95% CI)**	**p-value**	**HR (95% CI)**	**p-value**
4E-BP1	1.06 (0.86-1.3)	0.57	1.07 (0.86-1.34)	0.54	0.98 (0.78-1.23)	0.86	1.08 (0.87-1.34)	0.48
ADA	1.08 (0.64-1.82)	0.78	1.01 (0.57-1.81)	0.96	1.1 (0.62-1.96)	0.74	0.94 (0.45-1.93)	0.86
AXIN1	1.21 (0.95-1.52)	0.12	1.15 (0.92-1.43)	0.21	1.03 (0.81-1.31)	0.81	1.13 (0.9-1.42)	0.29
CASP-8	1.08 (0.8-1.46)	0.61	1.14 (0.67-1.93)	0.64	0.87 (0.52-1.46)	0.61	0.9 (0.5-1.62)	0.74
CCL11	1.48 (0.87-2.52)	0.14	0.89 (0.5-1.59)	0.70	1.22 (0.74-2.02)	0.43	0.86 (0.47-1.56)	0.61
CCL19	1.55 (1.2-2.01)	1e-03	1.08 (0.8-1.45)	0.61	1.17 (0.88-1.56)	0.27	1.23 (0.86-1.75)	0.26
CCL20	0.98 (0.78-1.24)	0.87	1.15 (0.85-1.56)	0.38	0.95 (0.71-1.28)	0.76	1.01 (0.76-1.35)	0.92
CCL23	0.77 (0.38-1.55)	0.46	1.26 (0.76-2.09)	0.36	0.89 (0.45-1.74)	0.73	0.82 (0.42-1.6)	0.56
CCL25	1.39 (0.82-2.35)	0.22	1.4 (0.85-2.29)	0.19	1.08 (0.74-1.56)	0.69	0.91 (0.56-1.45)	0.68
CCL28	1.41 (0.71-2.8)	0.32	0.79 (0.43-1.48)	0.47	1.02 (0.58-1.77)	0.96	0.76 (0.38-1.51)	0.43
CCL3	1.69 (0.98-2.89)	0.06	1.5 (0.94-2.39)	0.09	0.96 (0.58-1.61)	0.89	0.84 (0.45-1.57)	0.58
CCL4	0.97 (0.61-1.55)	0.91	1.01 (0.61-1.66)	0.98	0.8 (0.51-1.26)	0.33	0.85 (0.52-1.39)	0.52
CD244	1.59 (0.68-3.68)	0.28	1.33 (0.63-2.8)	0.45	0.92 (0.45-1.88)	0.82	0.61 (0.24-1.52)	0.28
CD40	1.98 (1.12-3.5)	0.02	1.24 (0.73-2.11)	0.43	1.03 (0.55-1.93)	0.92	1.06 (0.53-2.14)	0.86
CD5	1.21 (0.62-2.37)	0.57	0.78 (0.4-1.56)	0.49	1.09 (0.6-1.96)	0.79	0.58 (0.24-1.4)	0.22
CD6	0.73 (0.43-1.25)	0.25	0.96 (0.61-1.52)	0.87	1.2 (0.73-1.97)	0.47	0.52 (0.29-0.93)	0.03
CDCP1	1.48 (1.03-2.13)	0.04	1.03 (0.67-1.58)	0.91	1.23 (0.78-1.96)	0.37	1.58 (0.93-2.67)	0.09
hsCRP	1.18 (1.01-1.39)	0.04	1.23 (1.06-1.41)	5e-03	1.02 (0.86-1.2)	0.86	1.04 (0.89-1.22)	0.62
CSF-1	2.3 (1.02-5.2)	0.046	1.55 (0.68-3.52)	0.30	0.85 (0.38-1.93)	0.70	0.5 (0.19-1.33)	0.17
CST5	1.89 (1.14-3.13)	0.01	0.94 (0.5-1.76)	0.85	1.21 (0.73-1.99)	0.45	0.9 (0.48-1.7)	0.74
CX3CL1	1.17 (0.6-2.3)	0.65	1.06 (0.54-2.07)	0.87	0.77 (0.47-1.27)	0.31	1.12 (0.51-2.45)	0.78
CXCL1	1.34 (1-1.8)	0.053	1.01 (0.79-1.28)	0.95	0.93 (0.76-1.14)	0.49	0.85 (0.66-1.09)	0.19
CXCL10	1.66 (1.19-2.29)	3e-03	1.13 (0.85-1.5)	0.41	0.82 (0.6-1.12)	0.22	1.11 (0.79-1.55)	0.56
CXCL11	1.75 (1.21-2.51)	3e-03	1.2 (0.96-1.49)	0.11	0.84 (0.6-1.18)	0.31	0.81 (0.57-1.16)	0.25
CXCL5	1.2 (0.98-1.46)	0.08	1 (0.84-1.19)	0.98	0.98 (0.86-1.11)	0.75	0.89 (0.75-1.06)	0.20
CXCL6	1.59 (1.1-2.31)	0.01	1.21 (0.9-1.63)	0.20	0.84 (0.59-1.2)	0.34	0.78 (0.51-1.21)	0.27
CXCL9	1.39 (1.02-1.91)	0.04	1.04 (0.8-1.34)	0.79	0.85 (0.63-1.15)	0.30	0.97 (0.71-1.34)	0.87
DNER	0.48 (0.19-1.2)	0.12	0.31 (0.13-0.74)	8e-03	1.09 (0.47-2.54)	0.84	0.42 (0.15-1.19)	0.10
FGF-19	0.91 (0.68-1.2)	0.49	1.07 (0.8-1.44)	0.66	0.96 (0.74-1.26)	0.79	1.3 (0.88-1.91)	0.18
FGF-21	1 (0.8-1.24)	0.98	1.05 (0.86-1.3)	0.62	0.85 (0.69-1.06)	0.14	1 (0.78-1.28)	1.00
FGF-23	1.37 (0.92-2.04)	0.13	1.3 (0.95-1.77)	0.10	0.91 (0.56-1.47)	0.69	1.04 (0.62-1.76)	0.87
Flt3L	1.73 (0.96-3.13)	0.07	0.48 (0.29-0.77)	3e-03	1.13 (0.64-1.97)	0.68	0.78 (0.42-1.45)	0.43
hGDNF	1.48 (0.78-2.8)	0.23	0.89 (0.48-1.64)	0.70	1.44 (0.76-2.7)	0.26	0.91 (0.46-1.8)	0.78
HGF	1.09 (0.78-1.51)	0.62	0.99 (0.73-1.35)	0.96	0.89 (0.59-1.34)	0.58	1.04 (0.7. 1.53)	0.86

…

Grey shaded, nominally significant in one or more analyses; LAA, large artery atherosclerosis; CE, cardioembolic; SAO, small artery occlusion

Table S6, continued. Hazard ratios (HR) and 95% confidence intervals (CI) for MACE stratified by etiologic ischemic stroke subtype per doubling of protein levels from univariable and multivariable Cox regressions.

	Univariable							
	LAA		CE		SAO		Cryptogenic	
Protein	HR (95% CI)	p-value	HR (95% CI)	p-value	HR (95% CI)	p-value	HR (95% CI)	p-value
IFNgamma	2 (1.31-3.07)	1e-03	1.2 (0.83-1.73)	0.33	0.72 (0.42-1.23)	0.23	0.49 (0.2-1.19)	0.11
IL-10	1.2 (0.73-1.95)	0.47	1.43 (0.96-2.13)	0.08	1.22 (0.95-1.58)	0.13	0.76 (0.46-1.28)	0.30
IL-10RB	3.07 (1.33-7.1)	0.01	1.47 (0.7-3.11)	0.31	0.86 (0.49-1.49)	0.59	1.01 (0.46-2.21)	0.98
IL-12B	1.67 (1.08-2.6)	0.02	1.01 (0.68-1.5)	0.97	0.94 (0.65-1.34)	0.72	1.28 (0.81-2.02)	0.29
IL-18	1.24 (0.74-2.09)	0.42	1.31 (0.84-2.03)	0.24	1.09 (0.7-1.7)	0.70	0.82 (0.47-1.41)	0.47
IL-18R1	1.36 (0.68-2.72)	0.38	1.5 (0.83-2.68)	0.18	1 (0.51-1.98)	1.00	0.67 (0.33-1.34)	0.25
IL-6	1.24 (0.89-1.73)	0.21	1.25 (1.02-1.54)	0.03	1.19 (0.9-1.59)	0.23	1.03 (0.8-1.33)	0.81
IL-7	1.42 (0.99-2.04)	0.06	1.07 (0.79-1.46)	0.66	0.85 (0.58-1.23)	0.39	0.93 (0.61-1.42)	0.74
IL-8	1.25 (0.9-1.74)	0.18	1.15 (0.73-1.83)	0.55	1.19 (0.86-1.66)	0.29	1.01 (0.69-1.47)	0.98
LAP TGF-beta-1	1.64 (0.9-2.98)	0.11	1.09 (0.64-1.84)	0.76	0.94 (0.55-1.62)	0.83	0.71 (0.39-1.29)	0.26
LIF-R	0.63 (0.22-1.81)	0.39	1.15 (0.51-2.6)	0.73	1.13 (0.55-2.31)	0.73	0.49 (0.2-1.16)	0.10
MCP-1	1.8 (1.07-3.03)	0.03	1.09 (0.57-2.08)	0.79	1.18 (0.72-1.95)	0.51	0.77 (0.39-1.53)	0.46
MCP-2	1.53 (0.93-2.52)	0.10	0.89 (0.6-1.31)	0.55	1.09 (0.7-1.69)	0.70	0.69 (0.41-1.18)	0.18
MCP-3	1.31 (0.85-2.01)	0.22	1.41 (0.98-2.03)	0.07	1.42 (0.98-2.06)	0.06	1.12 (0.68-1.84)	0.66
MCP-4	1.39 (0.95-2.04)	0.09	0.76 (0.47-1.22)	0.25	0.9 (0.62-1.32)	0.60	1.11 (0.68-1.8)	0.69
MMP-10	0.98 (0.7-1.36)	0.89	0.91 (0.59-1.39)	0.65	1.19 (0.78-1.82)	0.41	0.96 (0.66-1.41)	0.85
NT-3	1.26 (0.92-1.74)	0.15	0.91 (0.48-1.72)	0.77	0.95 (0.51-1.78)	0.88	0.68 (0.34-1.35)	0.27
OPG	1.76 (0.86-3.63)	0.12	0.91 (0.48-1.73)	0.78	2.17 (1.1-4.31)	0.03	1.05 (0.49-2.26)	0.91
OSM	1.01 (0.77-1.33)	0.94	1.15 (0.88-1.51)	0.31	0.88 (0.66-1.17)	0.38	0.97 (0.73-1.3)	0.86
S100A12	1.33 (1-1.78)	0.052	1.38 (1.09-1.75)	8e-03	1.68 (1.21-2.34)	3e-03	1.19 (0.79-1.79)	0.41
SCF	0.97 (0.58-1.63)	0.91	0.74 (0.45-1.23)	0.25	0.73 (0.4-1.32)	0.30	0.63 (0.35-1.14)	0.13
SIRT2	1.08 (0.9-1.3)	0.42	1.1 (0.9-1.35)	0.33	0.97 (0.78-1.21)	0.80	1.06 (0.87-1.29)	0.57
SLAMF1	1.03 (0.62-1.69)	0.92	1.4 (0.91-2.13)	0.12	0.96 (0.6-1.54)	0.87	0.94 (0.46-1.89)	0.86
STAMBP	1.15 (0.89-1.48)	0.27	1.17 (0.91-1.52)	0.22	0.99 (0.73-1.33)	0.94	1.1 (0.84-1.43)	0.50
TNFB	1.4 (0.98-1.99)	0.06	1.05 (0.61-1.8)	0.87	1.08 (0.65-1.79)	0.76	0.7 (0.39-1.26)	0.24
TNFRSF9	1.9 (1.08-3.34)	0.03	1.5 (0.81-2.79)	0.20	1.03 (0.62-1.71)	0.90	0.88 (0.44-1.76)	0.72
TNFSF14	1.33 (0.8-2.22)	0.27	1.73 (1-2.98)	0.0496	0.95 (0.51-1.78)	0.87	1.03 (0.55-1.92)	0.93
TRAIL	0.93 (0.44-1.99)	0.86	0.84 (0.45-1.58)	0.59	0.83 (0.35-1.94)	0.67	0.35 (0.17-0.76)	0.01
TRANCE	0.73 (0.49-1.08)	0.12	1.03 (0.68-1.56)	0.88	0.73 (0.48-1.12)	0.15	0.85 (0.56-1.29)	0.44
TWEAK	0.72 (0.38-1.38)	0.32	0.63 (0.36-1.1)	0.10	0.81 (0.43-1.52)	0.51	0.69 (0.36-1.31)	0.26
uPA	1.18 (0.55-2.56)	0.67	0.99 (0.52-1.88)	0.97	1.02 (0.47-2.18)	0.97	0.77 (0.3-2.02)	0.60
VEGFA	2.35 (1.3-4.26)	5e-03	1.52 (0.78-2.94)	0.22	1.07 (0.59-1.95)	0.81	1.13 (0.53-2.39)	0.76

Grey shaded, nominally significant in one or more analyses; LAA, large artery atherosclerosis; CE, cardioembolic; SAO, small artery occlusion

Table S6, continued. Hazard ratios (HR) and 95% confidence intervals (CI) for MACE stratified by etiologic ischemic stroke subtype per doubling of protein levels from univariable and multivariable Cox regressions.

	**Adjusted for: age, sex, day of blood draw, diabetes, hypertension, previous CAD, statin treatment**							
	**LAA**		**CE**		**SAO**		**Cryptogenic**	
**Protein**	**HR (95% CI)**	**p-value**	**HR (95% CI)**	**p-value**	**HR (95% CI)**	**p-value**	**HR (95% CI)**	**p-value**
4E-BP1	0.93 (0.73-1.19)	0.58	1.15 (0.89-1.50)	0.28	1.06 (0.83-1.36)	0.62	1.05 (0.84-1.32)	0.67
ADA	1.25 (0.70-2.21)	0.45	1.25 (0.69-2.29)	0.46	1.18 (0.65-2.17)	0.58	0.91 (0.42-1.95)	0.80
AXIN1	1.06 (0.81-1.39)	0.68	1.19 (0.94-1.50)	0.16	1.04 (0.80-1.34)	0.79	1.10 (0.87-1.40)	0.41
CASP-8	1.00 (0.68-1.48)	0.99	1.42 (0.82-2.45)	0.21	1.10 (0.61-1.98)	0.74	0.86 (0.47-1.60)	0.64
CCL11	1.78 (0.89-3.55)	0.10	0.80 (0.41-1.56)	0.52	1.37 (0.75-2.49)	0.30	0.61 (0.32-1.18)	0.14
CCL19	1.39 (0.97-2.01)	0.08	1.12 (0.81-1.55)	0.50	1.14 (0.83-1.57)	0.42	1.23 (0.83-1.81)	0.31
CCL20	0.89 (0.64-1.24)	0.50	1.20 (0.84-1.73)	0.31	0.96 (0.70-1.33)	0.81	0.97 (0.73-1.28)	0.82
CCL23	0.68 (0.26-1.76)	0.42	1.40 (0.77-2.55)	0.27	0.77 (0.37-1.58)	0.47	0.73 (0.35-1.53)	0.41
CCL25	1.40 (0.82-2.39)	0.21	1.34 (0.72-2.49)	0.36	1.02 (0.68-1.55)	0.91	0.80 (0.51-1.27)	0.34
CCL28	1.88 (0.83-4.25)	0.13	0.78 (0.38-1.58)	0.49	0.91 (0.47-1.79)	0.79	0.60 (0.25-1.45)	0.26
CCL3	1.46 (0.72-2.99)	0.30	1.62 (0.89-2.92)	0.11	0.96 (0.54-1.71)	0.90	0.76 (0.39-1.46)	0.41
CCL4	1.18 (0.64-2.14)	0.60	1.18 (0.66-2.12)	0.58	0.90 (0.55-1.46)	0.67	0.87 (0.51-1.49)	0.60
CD244	1.43 (0.53-3.81)	0.48	1.48 (0.64-3.38)	0.36	0.96 (0.44-2.11)	0.92	0.60 (0.23-1.60)	0.31
CD40	1.98 (0.98-4.02)	0.06	1.31 (0.76-2.26)	0.33	0.87 (0.44-1.73)	0.69	0.94 (0.45-1.95)	0.87
CD5	0.94 (0.44-2.03)	0.88	0.68 (0.29-1.57)	0.37	0.99 (0.50-1.96)	0.98	0.66 (0.28-1.55)	0.33
CD6	0.51 (0.27-0.98)	0.04	1.00 (0.60-1.67)	0.99	1.27 (0.74-2.18)	0.38	0.50 (0.28-0.90)	0.02
CDCP1	1.68 (1.03-2.73)	0.04	0.89 (0.52-1.53)	0.68	1.14 (0.66-1.95)	0.65	1.10 (0.59-2.07)	0.76
hsCRP	1.13 (0.94-1.35)	0.20	1.32 (1.10-1.59)	2.8e-03	0.95 (0.80-1.13)	0.57	0.95 (0.79-1.15)	0.62
CSF-1	3.65 (1.21-11.00)	0.02	1.91 (0.74-4.89)	0.18	0.72 (0.28-1.82)	0.48	0.41 (0.15-1.14)	0.09
CST5	2.02 (1.15-3.56)	0.01	0.85 (0.43-1.71)	0.66	1.04 (0.61-1.78)	0.88	0.77 (0.41-1.46)	0.43
CX3CL1	1.29 (0.63-2.63)	0.49	1.02 (0.47-2.20)	0.96	0.64 (0.36-1.15)	0.14	0.97 (0.43-2.15)	0.93
CXCL1	1.49 (1.01-2.18)	0.04	1.09 (0.85-1.41)	0.48	0.96 (0.77-1.19)	0.71	0.81 (0.61-1.07)	0.14
CXCL10	1.76 (1.16-2.66)	7.4e-03	1.12 (0.80-1.57)	0.50	0.80 (0.57-1.11)	0.18	1.04 (0.73-1.50)	0.82
CXCL11	1.87 (1.17-2.98)	9.1e-03	1.23 (0.97-1.56)	0.09	0.84 (0.60-1.18)	0.32	0.81 (0.57-1.17)	0.26
CXCL5	1.13 (0.92-1.40)	0.24	1.11 (0.90-1.37)	0.31	0.98 (0.85-1.12)	0.75	0.88 (0.73-1.06)	0.19
CXCL6	1.51 (0.99-2.29)	0.05	1.25 (0.94-1.68)	0.13	0.81 (0.55-1.20)	0.29	0.80 (0.50-1.26)	0.33
CXCL9	1.60 (1.09-2.35)	0.02	0.95 (0.68-1.32)	0.76	0.76 (0.54-1.07)	0.11	0.91 (0.63-1.31)	0.61
DNER	0.99 (0.34-2.89)	0.98	0.31 (0.11-0.89)	0.03	1.37 (0.52-3.61)	0.52	0.30 (0.09-1.00)	0.05
FGF-19	0.91 (0.68-1.22)	0.52	1.03 (0.76-1.40)	0.84	1.02 (0.76-1.36)	0.90	1.29 (0.88-1.89)	0.20
FGF-21	1.24 (0.93-1.65)	0.14	1.03 (0.80-1.32)	0.83	0.85 (0.66-1.10)	0.22	0.85 (0.63-1.15)	0.29
FGF-23	1.22 (0.72-2.07)	0.47	1.33 (0.92-1.92)	0.13	0.95 (0.54-1.66)	0.86	0.87 (0.52-1.45)	0.58
Flt3L	1.98 (0.96-4.07)	0.06	0.39 (0.22-0.70)	1.6e-03	1.00 (0.55-1.80)	0.99	0.55 (0.28-1.07)	0.08
hGDNF	1.87 (0.77-4.54)	0.17	0.93 (0.45-1.94)	0.84	1.47 (0.74-2.93)	0.27	0.63 (0.27-1.47)	0.28
HGF	1.34 (0.85-2.09)	0.21	1.05 (0.75-1.47)	0.78	0.95 (0.60-1.49)	0.81	0.99 (0.61-1.61)	0.98

…

Grey shaded, nominally significant in one or more analyses; LAA, large artery atherosclerosis; CE, cardioembolic; SAO, small artery occlusion

Table S6, continued. Hazard ratios (HR) and 95% confidence intervals (CI) for MACE stratified by etiologic ischemic stroke subtype per doubling of protein levels from univariable and multivariable Cox regressions.

	Adjusted for: age, sex, day of blood draw, diabetes, hypertension, previous CAD, statin treatment							
	LAA		CE		SAO		Cryptogenic	
**Protein**	**HR (95% CI)**	**p-value**	**HR (95% CI)**	**p-value**	**HR (95% CI)**	**p-value**	**HR (95% CI)**	**p-value**
IFNgamma	2.03 (1.24-3.31)	4.7e-03	1.24 (0.79-1.92)	0.35	0.70 (0.39-1.27)	0.24	0.47 (0.19-1.19)	0.11
IL-10	1.76 (0.99-3.13)	0.06	1.75 (1.08-2.81)	0.02	1.22 (0.94-1.59)	0.13	0.80 (0.49-1.31)	0.37
IL-10RB	1.94 (0.70-5.37)	0.20	1.35 (0.57-3.19)	0.50	0.83 (0.46-1.50)	0.54	0.89 (0.41-1.94)	0.77
IL-12B	1.55 (0.94-2.55)	0.09	0.91 (0.59-1.41)	0.68	0.73 (0.47-1.13)	0.16	1.41 (0.86-2.31)	0.18
IL-18	2.02 (0.95-4.31)	0.07	1.52 (0.93-2.48)	0.10	1.07 (0.67-1.69)	0.78	0.78 (0.43-1.42)	0.42
IL-18R1	1.90 (0.77-4.65)	0.16	1.36 (0.67-2.74)	0.39	0.83 (0.37-1.88)	0.66	0.52 (0.26-1.06)	0.07
IL-6	1.18 (0.79-1.76)	0.43	1.36 (1.06-1.75)	0.02	1.13 (0.82-1.55)	0.46	0.94 (0.72-1.23)	0.67
IL-7	1.21 (0.79-1.84)	0.39	1.15 (0.81-1.64)	0.43	0.88 (0.60-1.30)	0.53	0.93 (0.61-1.43)	0.74
IL-8	1.38 (0.94-2.02)	0.10	1.40 (0.81-2.44)	0.23	1.27 (0.85-1.90)	0.24	0.86 (0.57-1.31)	0.48
LAP TGF-beta-1	1.17 (0.59-2.32)	0.65	1.07 (0.62-1.84)	0.80	1.00 (0.57-1.75)	0.99	0.67 (0.33-1.35)	0.26
LIF-R	1.38 (0.40-4.78)	0.61	1.21 (0.49-3.01)	0.68	1.03 (0.45-2.33)	0.95	0.27 (0.10-0.68)	5.7e-03
MCP-1	2.06 (1.08-3.93)	0.03	0.96 (0.46-1.99)	0.91	1.12 (0.65-1.93)	0.68	0.58 (0.28-1.21)	0.15
MCP-2	1.38 (0.78-2.46)	0.27	0.86 (0.56-1.34)	0.52	1.12 (0.69-1.80)	0.65	0.53 (0.30-0.95)	0.03
MCP-3	1.28 (0.74-2.21)	0.38	1.29 (0.82-2.03)	0.28	1.55 (1.00-2.40)	0.05	0.98 (0.54-1.75)	0.93
MCP-4	1.22 (0.75-1.99)	0.42	0.56 (0.32-0.98)	0.04	0.82 (0.54-1.23)	0.33	1.04 (0.63-1.73)	0.88
MMP-10	0.91 (0.63-1.30)	0.60	0.86 (0.53-1.38)	0.53	1.32 (0.79-2.19)	0.29	0.96 (0.64-1.45)	0.85
NT-3	0.87 (0.56-1.33)	0.51	1.12 (0.58-2.16)	0.74	1.17 (0.61-2.26)	0.64	0.71 (0.36-1.38)	0.31
OPG	1.36 (0.61-3.01)	0.45	0.63 (0.29-1.35)	0.24	1.95 (0.85-4.50)	0.12	0.69 (0.28-1.71)	0.42
OSM	0.94 (0.68-1.29)	0.69	1.21 (0.89-1.64)	0.23	0.99 (0.74-1.34)	0.96	0.93 (0.68-1.28)	0.67
S100A12	1.66 (1.05-2.61)	0.03	1.34 (1.01-1.80)	0.049	1.64 (1.15-2.33)	5.8e-03	1.21 (0.75-1.94)	0.43
SCF	1.02 (0.54-1.94)	0.94	0.62 (0.35-1.09)	0.10	0.65 (0.34-1.24)	0.19	0.54 (0.29-1.00)	0.049
SIRT2	0.96 (0.76-1.20)	0.70	1.18 (0.94-1.48)	0.15	1.02 (0.81-1.29)	0.88	1.04 (0.84-1.28)	0.72
SLAMF1	0.94 (0.51-1.74)	0.85	1.35 (0.83-2.19)	0.23	0.91 (0.53-1.56)	0.73	0.78 (0.37-1.65)	0.51
STAMBP	0.99 (0.73-1.35)	0.95	1.28 (0.97-1.70)	0.08	1.05 (0.76-1.45)	0.76	1.06 (0.80-1.41)	0.67
TNFB	1.45 (0.94-2.23)	0.10	1.10 (0.59-2.06)	0.76	1.08 (0.62-1.88)	0.79	0.76 (0.40-1.47)	0.42
TNFRSF9	1.75 (0.95-3.23)	0.08	1.43 (0.70-2.90)	0.32	0.87 (0.49-1.52)	0.62	0.83 (0.40-1.73)	0.61
TNFSF14	1.67 (0.83-3.38)	0.15	1.93 (1.08-3.45)	0.03	0.97 (0.50-1.88)	0.94	0.93 (0.46-1.87)	0.84
TRAIL	1.01 (0.47-2.15)	0.99	0.89 (0.42-1.89)	0.75	0.98 (0.41-2.37)	0.97	0.32 (0.15-0.70)	4.2e-03
TRANCE	0.90 (0.57-1.43)	0.67	1.09 (0.68-1.75)	0.71	0.84 (0.52-1.36)	0.48	0.85 (0.57-1.28)	0.44
TWEAK	0.99 (0.47-2.10)	0.99	0.61 (0.32-1.16)	0.13	0.77 (0.39-1.52)	0.45	0.68 (0.33-1.38)	0.28
uPA	1.36 (0.55-3.34)	0.51	0.95 (0.48-1.86)	0.88	0.88 (0.38-2.01)	0.76	0.78 (0.31-1.96)	0.60
VEGFA	1.96 (0.92-4.16)	0.08	1.80 (0.84-3.83)	0.13	1.10 (0.58-2.08)	0.77	0.80 (0.34-1.86)	0.61

Grey shaded, nominally significant in one or more analyses; LAA, large artery atherosclerosis; CE, cardioembolic; SAO, small artery occlusion

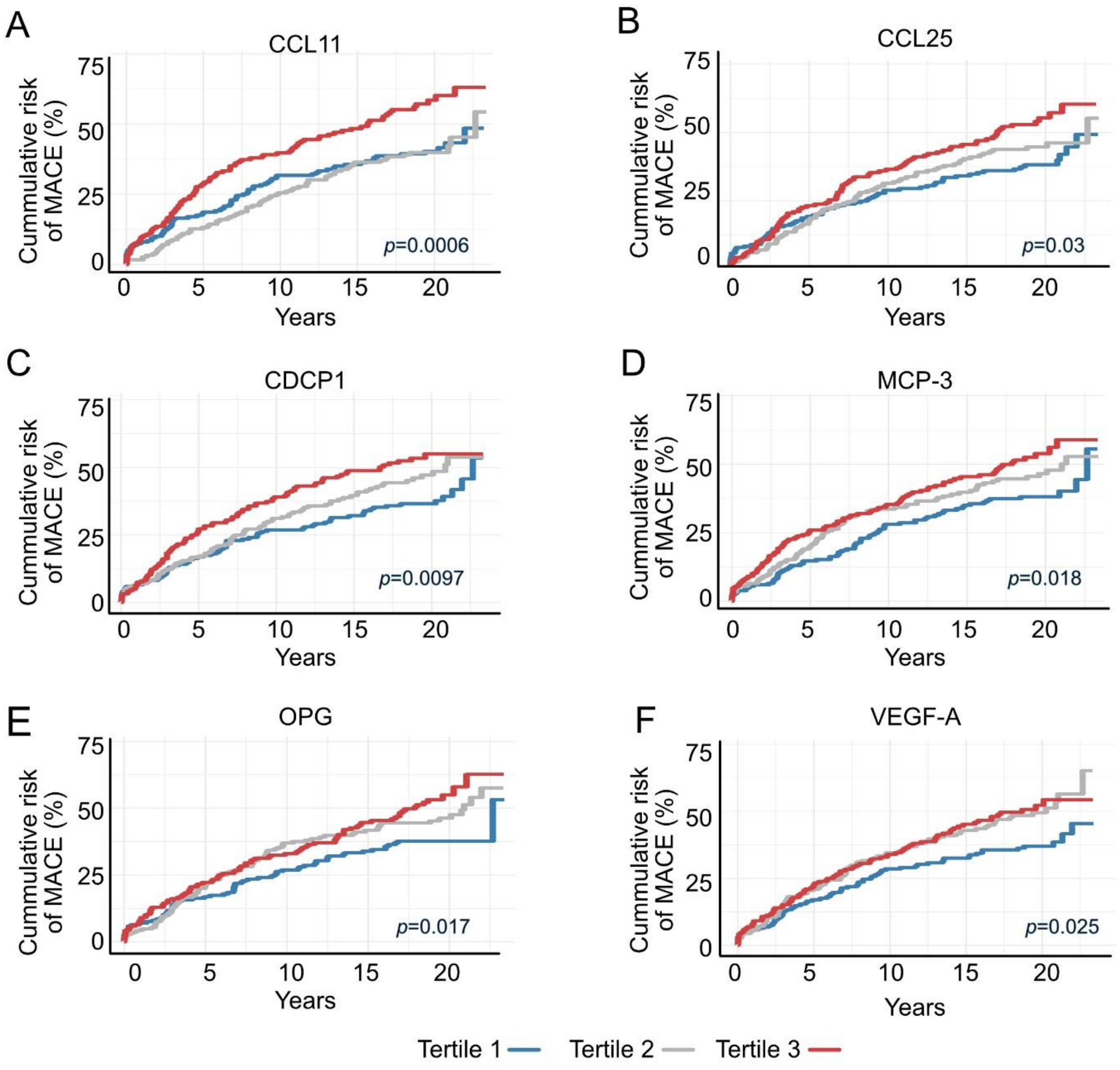

Figure S1. Kaplan-Meier curves showing cumulative incidence of MACE for protein tertiles in *SAHLSIS*. A) CCL11, B) CCL25, C) CDCP1, D) MCP-3 [CCL7], E) OPG [TNFRSF11B], and F) VEGF-A. Blue, tertile 1 (lowest protein levels); grey, tertile 2; red, tertile 3 (highest protein levels). Differences in cumulative incidence of recurrent MACE between tertile groups were assessed by standard log-rank tests, with p-values < 0.05 considered significant.
